# 3D-Printed Polycaprolactone/Alginate-Whitlockite Composite Scaffolds for Bioactive Bone Tissue Engineering

**DOI:** 10.1021/acsmaterialsau.6c00160

**Published:** 2026-10-08

**Authors:** Sura Ali Al-nidawi, Aliakbar Najafinezhad, Hossein Abbastabar Ahangar, Hamid Reza Bakhsheshi-Rad, Jaroslaw W. Drelich

**Affiliations:** † Department of Materials Engineering, Na.C., 201564 Islamic Azad University, Najafabad , Iran; ‡ Department of Chemistry, Na.C., 201564 Islamic Azad University, Najafabad , Iran; § Department of Materials Science and Engineering, 3968 Michigan Technological University, Houghton, Michigan 49931, United States; ∥ Faculty of Mechanical Engineering, Universiti Teknologi Malaysia, 81310 UTM Johor Bahru, Johor, Malaysia

**Keywords:** 3D printing scaffold, alginate, whitlockite, mechanical properties, biocompatibility, bioactivity, bone tissue engineering

## Abstract

Despite advances in 3D printing, achieving simultaneous mechanical stability and biological functionality in polymer-based scaffolds remains challenging for bone tissue engineering applications. In this study, 3D-printed scaffolds fabricated from a polycaprolactone/alginate (PCL/Alg) composite reinforced with different amounts of whitlockite (WL) nanoparticles (including 0, 5, 10, 20, and 30 wt %) were designed. Mechanical studies showed that adding 5 wt % WL significantly improved the mechanical properties, with the compressive strength and elastic modulus increasing from about 7 and 12 MPa to 22 and 48 MPa, respectively. However, increasing the WL content beyond this value reduced mechanical performance, likely due to WL particle aggregation and disruption of the PCL/Alg scaffold. Water contact angle analysis showed that the PCL/Alg scaffold incorporated with WL exhibited better hydrophilicity than pure PCL and PCL/Alg, which was mainly attributed to the hydrophilic nature of these compounds. Immersion results indicated that adding WL facilitated apatite deposition, mainly through the release of calcium and magnesium ions. The MTT assay showed that none of the designed scaffolds was toxic to MG-63 cells. Meanwhile, the scaffold containing 5 wt % WL exhibited the highest cell viability and adhesion, whereas higher WL concentrations yielded less favorable cellular responses. Taken together, the results obtained indicate that the PCL/Alg scaffold containing 5% WL provides a balanced combination of mechanical and biological properties, making it a desirable bioactive platform for bone tissue engineering applications.

## Graphical abstract

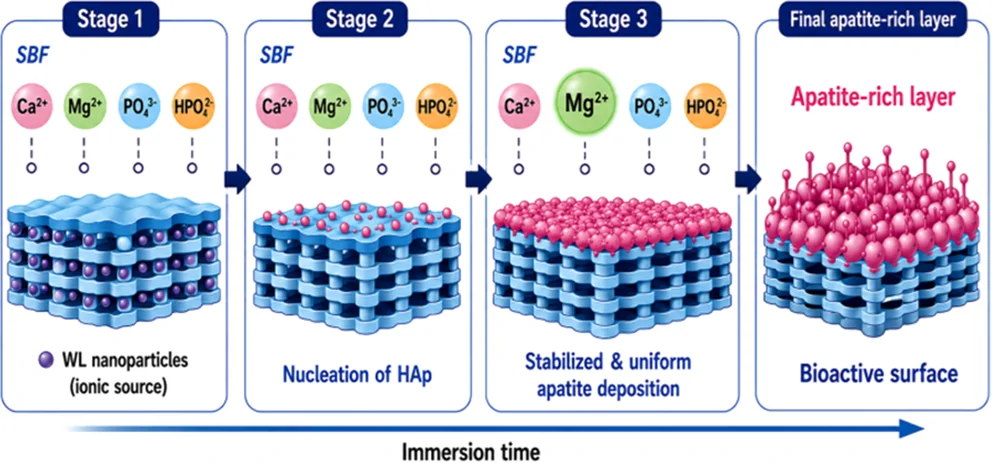

## 1 Introduction

One of the fundamental goals of tissue engineering is to restore the normal function of damaged or degraded tissues, a process pursued through a combination of biocompatible and biodegradable materials, living cells, and biological signaling factors.^1,2^ Within this framework, the focus is on developing systems that not only have the ability to replace tissue, but also provide suitable conditions for active repair and targeted regeneration.^3,4^ One of the key factors for success in this field is the careful selection of the biomaterial type used to construct scaffolds, especially those that can mimic the structure and function of the tissue’s native extracellular matrix (ECM) as closely as possible.^5,6^ In bone, the ECM exhibits a complex arrangement of collagen fibers and minerals, organized at multiple levels, that plays a fundamental role in maintaining mechanical function.^4,7^ Despite extensive studies on various materials for constructing bone scaffolds, most efforts have focused on matching the chemical composition to bone and providing structural strength.^8^ However, limitations such as poor porosity design, poor mechanical properties, and difficulty in producing uniform scaffolds still persist.^8–10^ Therefore, developing scaffolds that are not only biocompatible and biodegradable but also mimic the complex, multilayered structure of natural bone tissue remains one of the main challenges in this field.^2,7,10^ For this reason, bioceramics and engineered polymers with osteogenic, bioactive, and biodegradable properties have attracted researchers’ attention as a promising approach.^2,4^ Among synthetic polymers, PCL is a popular choice for manufacturing hard tissue scaffolds due to its high biocompatibility, controllable degradation rate, desirable physical strength, and the ability to be fabricated in various formats.^11–13^ However, problems such as low water affinity, poor cell adhesion, and the potential to generate acidic degradation products limit its use. Therefore, combining PCL with natural polymers or adding bionanoparticles to enhance its biological properties is a common strategy in designing more efficient scaffolds.^14–16^ In tissue engineering, Alg is widely considered a natural biopolymer because of its favorable compatibility with body tissues, biodegradability, low immune response, high gelling ability, and structural similarity to the extracellular matrix.^17–19^ However, pure Alg has limited application due to low mechanical strength and difficulty maintaining a stable three-dimensional structure, especially under applied conditions.^20–22^ To overcome these limitations, Alg incorporation with PCL, which exhibits suitable physical properties and high processability, is proposed. In such hybrid systems, Alg improves biocompatibility and hygroscopicity, while PCL plays a key role in enhancing mechanical properties and structural stability.^23–25^ Also, the incorporation of bioceramic nanoparticles into PCL/Alg system can facilitate the formation of mineral and apatite layers. Among bioceramics, WL exhibited better performance in inducing bone regeneration than other calcium phosphates, such as hydroxyapatite (HAp) and β-TCP, due to its specific chemical composition, which includes Mg^2+^ ions in the crystal structure.^26–28^ This magnesium ion, a large part of which is stored in bone tissue, plays a key role in regulating the proper functioning of the skeletal, muscular and nervous systems.^29,30^ In addition, WL exhibited greater ability to stimulate stem cell proliferation and differentiation in low-pH environments, making it a suitable candidate for use in bone regeneration scaffolds.^26,31^ The addition of WL to PCL-Alg scaffolds is considered a promising strategy to enhance biological function and induce osteogenesis. Unlike other common forms of calcium phosphate, this mineral compound is naturally found in the collagenous matrix of bone, alongside hydroxyapatite, and plays a key role, especially in the early stages of bone tissue development.^32,33^ The chemical structure of whitlockite, with the presence of magnesium and calcium ions, is distinct from β-TCP, and this difference is of great importance for its biological activity.^34–36^ As a vital element, magnesium plays an active role in bone metabolism, tissue mineralization, and biointegration. In addition, its high affinity for cell adhesion-related proteins, such as integrins, indicates its versatile role in tissue regeneration.^37–39^ From biological and physical perspectives, whitlockite exhibits desirable properties, including biocompatibility, bioactivity, biodegradability, high absorbability, and a negative surface charge.^40–42^ This combination of properties makes WL an ideal component for incorporation into polymer-based scaffolds; PCL provides the necessary structure and strength, Alg enhances biological responses and hydrophilicity, and WL enhances mineralization potential and effective interaction with bone cells.^22,28^ Despite these advantages, few studies have investigated the performance of WL in 3D-printed scaffolds composed of PCL and Alg.^17,19,32,34^ Therefore, the present study focuses on the development and evaluation of a 3D-printed PCL/Alg-WL based composite scaffold to create a functional platform for bone tissue engineering.

## 2 Materials and Methods

### 2.1 Materials

Poly­(ε-caprolactone) (PCL, average Mn = 80 000 g/mol; Sigma-Aldrich, USA) and sodium alginate (SA; LV Extrapure, 4–12 cP, SRL, India) were used as polymeric components. Dicalcium phosphate dihydrate (CaHPO_4_·2H_2_O), magnesium acetate tetrahydrate (Mg­(CH_3_COO)_2_·4H_2_O), and phosphoric acid (H_3_PO_4_) were used as precursors for the synthesis of whitlockite (WL) nanoparticles and were obtained from Merck (Darmstadt, Germany). Deionized distilled water was used to prepare the solutions. Chloroform, dimethyl sulfoxide (DMSO), and ethanol were purchased from Merck (Darmstadt, Germany).

### 2.2 Synthesis of WL Nanoparticles

To prepare whitlockite (WL; Ca_18_Mg_2_(HPO_4_)_2_(PO_4_)_12_), a solution precipitation method was used.^43,44^ were employed as the main sources of calcium, phosphate, and magnesium ions. In this process, dicalcium phosphate dihydrate (CaHPO_4_·2H_2_O) and magnesium acetate tetrahydrate (Mg­(CH_3_COO)_2_·4H_2_O) were dissolved in 100 mL of deionized distilled water; the mixture was heated to 65 °C, and 11 mL of 1 M H_3_PO_4_ was subsequently added. After the solution became clear at approximately pH 2.7, the reaction was maintained for 30 min, followed by dropwise addition of 25% aqueous ammonia until the pH reached 5.6. At this stage, WL precipitate began to form, a process shown in Figure 1a. The resulting suspension was maintained under continuous stirring for 24 h to allow the synthesis and aging process to proceed. The precipitate was then collected, washed, and dried at 60 °C for 24 h. The precipitate was separated from the solution and washed several times with distilled water to completely remove unreacted ions and impurities. The dried powder was subsequently collected for characterization and scaffold fabrication.

**Figure 1:**
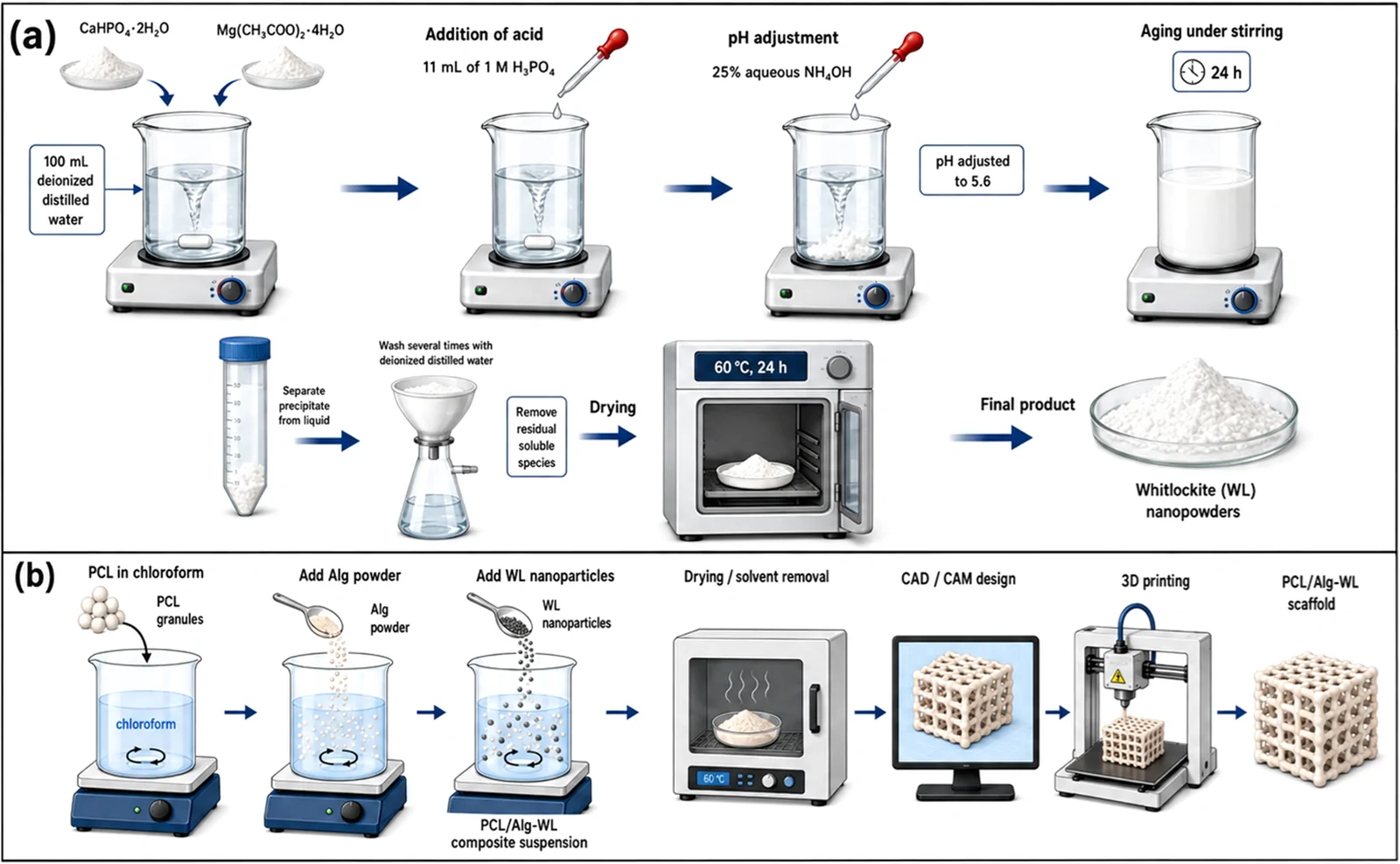
Schematic illustration of (a) synthesis process of whitlockite, and (b) 3D-printed PCL/Alg-WL composite scaffolds, including preparation of the PCL/Alg-WL mixture, incorporation of WL nanoparticles, solvent removal, and subsequent FDM-based 3D printing.

### 2.3 Fabrication of 3D-Printed PCL/Alg-WL Scaffolds

In this study, multicomponent PCL/Alg-WL scaffolds were fabricated using a desktop 3D bioprinter (Chakad, Iran) via a melt-extrusion-based additive manufacturing approach, as illustrated in Figure 1b. A computer-aided design/computer-aided manufacturing (CAD/CAM) strategy was employed to construct the three-dimensional scaffold architecture through controlled layer-by-layer deposition. The total mass of each formulation was maintained at 5.00 g, while the Alg content was fixed at 0.5 g, corresponding to 10 wt % of the total formulation. WL nanoparticles were incorporated at 0, 5, 10, 20, and 30 wt % relative to the total PCL/Alg/WL formulation, corresponding to WL masses of 0, 0.25, 0.5, 1, and 1.5 g, respectively. Accordingly, the PCL contents were adjusted to 4.5, 4.25, 4, 3.5, and 3 g, respectively, as summarized in Table S1. The Alg content was selected based on preliminary processing considerations, as higher Alg contents increased mixture viscosity and reduced processability, whereas 0.5 g Alg provided suitable handling and scaffold-fabrication characteristics.

For preparation of the composite feedstock, the required amount of PCL for each formulation was first dissolved in 10 mL of chloroform under magnetic stirring at 200 rpm until a homogeneous polymer solution was obtained. Because Alg is insoluble in chloroform, therefore, in the present preparation, only PCL was dissolved in chloroform, whereas Alg and WL were incorporated as dispersed solid phases within the PCL solution. This approach is consistent with related PCL/Alg processing strategies in which alginate is incorporated as a dispersed phase within the PCL matrix rather than molecularly dissolved in the PCL phase. For example, the referenced PCL/Alg studies incorporated alginate powder into molten PCL and subsequently processed the resulting composite mixture to obtain a homogeneous distribution. WL nanoparticles were subsequently incorporated at the designated concentrations, and the resulting PCL/Alg-WL mixture was continuously stirred for 24 h to promote uniform distribution of the constituent phases. Following mixing, the composite mixture was dried in an oven at 40 °C for 24 h to remove chloroform and obtain a dry PCL/Alg-WL composite feedstock. The dried composite was then processed by melt-extrusion-based 3D printing using a 400 μm nozzle, an extrusion temperature of 100 °C, a feed rate of 100%, and a nozzle speed of 100 mm/min. These parameters enabled stable extrusion and controlled layer-by-layer scaffold fabrication.

### 2.4 Microstructure, Morphology, and Crystalline Phase

Transmission electron microscopy (TEM; Zeiss-EM10C, Germany) was used to analyze the size distribution and morphological properties of the synthesized WL nanoparticles. The surface structure and microstructure of the 3D-printed scaffolds were also examined using a scanning electron microscope (SEM; FEI Quanta 450, USA). The spatial distribution of WL nanoparticles in the PCL/Alg composite framework was evaluated using energy-dispersive X-ray spectroscopy (EDS) performed simultaneously with SEM. The size of the nanoparticles, the thickness of the structural layers, and the diameter of the channels were measured using ImageJ software. Fourier transform infrared spectroscopy (FTIR; Nicolet 5700) was used to identify the chemical functional groups in the structure of WL nanoparticles and the resulting scaffolds. Spectra were collected in the range of 400–4000 cm^–1^ with a spectral resolution of 4 cm^–1^ and the data were analyzed using Origin software. The phase composition and crystal structure of the materials were also evaluated using X-ray diffraction (XRD; Philips X’Pert MPD). XRD patterns were recorded in the 2θ angular range of 10 to 90° with an angular step of 0.1° and the crystallite size of WL was estimated using the Scherrer equation.

1 $$\tau =\frac{K\lambda }{\beta \cdot \cos\theta }$$

In the analysis performed using the Scherrer equation, the X-ray wavelength was assumed to be 0.154 nm and the shape constant (*K*) was set to 1. The peak width at half height (β), expressed in radians, along with the Bragg diffraction angle (θ), was used as input to estimate the crystallite size. Since surface wettability plays a fundamental role in the initial interactions between the biological surface and the cellular environment, including cell adhesion, the water contact angle of the scaffolds was investigated. These measurements were performed using a contact angle determination device (model CA-X50 manufactured by FACE). In each test, a 4 μL droplet was carefully applied to the sample surface, and the corresponding image was recorded 10 s after contact. To increase the accuracy of the data, the experiments were repeated three to four times for each sample, and finally, the average results from three independent samples (*n* = 3) were reported.

### 2.5 Mechanical Properties and Degradation Rate

The compressive properties of the fabricated scaffolds were evaluated using specimens measuring 10 × 10 × 5 mm^3^, with reference to ASTM F2150-19. Compression testing was performed using an Instron 5566 universal testing machine (Darmstadt, Germany) equipped with a 10 kN load cell. The tests were conducted at 25 ± 1 °C with a constant crosshead speed of 1 mm/min. The recorded stress–strain curves were used to determine the compressive strength, and the slope of the initial linear portion of the curves was used to calculate the elastic modulus. To assess accuracy and reproducibility, three independent samples of each scaffold type were tested. The final results were reported as the average with standard deviation to ensure that the data reflected the system’s actual behavior.

To investigate the biostability of the scaffolds in vitro, a degradation test was performed in phosphate-buffered saline (PBS) over predetermined immersion periods of 1, 3, 7, 14, 21, 28, 35, 42, 49, and 56 days. The scaffolds were cut into 2.5 × 5 × 5 mm^3^ dimensions, and their initial dry weight (*W*_1_) was recorded. These samples were then placed in PBS to be exposed to simulated physiological conditions. At specific time intervals, the scaffolds were removed from the solution and washed with distilled water to remove any residual salt or soluble degradable materials. Then, the samples were subjected to vacuum drying conditions to accurately measure the final dry weight (*W*_2_). For each scaffold type, this process was performed with three independent replicates to maintain the statistical validity of the data. Finally, the weight degradation percentage of the scaffolds was calculated using the following equation:

2 $$\mathrm{Weight loss}=\frac{W_{1}-W_{2}}{W_{1}}$$

### 2.6 Mineralization Examination

To investigate the biomineralization potential of the scaffolds, the samples were incubated in 10 mL of SBF for 28 days under controlled temperature conditions (37 °C) with chemical composition as listed in Table S2. After this period, the scaffolds were removed from the reaction medium and rinsed with distilled water, then dried in an oven at 40 °C for 24 h. To study the surface morphology and distribution of mineral deposits, SEM images were used to investigate mineral growth and distribution on the scaffold surface. EDS analysis was also used to determine the chemical composition of the deposited layers.

### 2.7 Cell Cytotoxicity Assay and Adhesion

Before performing biological experiments, including cell culture, the scaffolds were sterilized in two steps: first, the samples were immersed in 70% ethanol for 30 min, and then each side of each sample was exposed to ultraviolet light for half an hour. Then, in order to remove residual solvent and possible materials, a three-step wash with sterile PBS was performed, and the samples were dried under sterile conditions. To investigate biocompatibility, the MTT assay was used using the MG-63 osteoblast-like cells (Institute Pasteur Cell Bank, Iran), which is known as an osteoblast-like model. In this regard, the cells were cultured at a density of 50 thousand cells per well in 12-well plates. The culture medium consisted of DMEM containing 10% fetal bovine serum and 1% antibiotic (penicillin-streptomycin). Cells were incubated at 37 °C and 5% CO_2_ for 24 h to allow for initial adhesion. After this step, the sterilized samples were transferred into the wells. At the desired times, MTT solution at a concentration of 5 mg/mL was added to the wells, and the samples were incubated for 3 h. After removal of the solution, the resulting crystals were dissolved in 100 μL of DMSO to obtain a purple color. The optical absorbance of the resulting solution was measured using an ELISA reader (model TS800, manufactured by Agilent/BioTek, USA). The increase in absorbance was considered as an indication of increased metabolic activity and the number of viable cells. The cell viability rate was calculated according to a specific formula.

3 $$\mathrm{Viability}\;\left(\%\right)=\frac{\mathrm{Mean OD of sample}}{\mathrm{Mean OD of control}}\times 100$$

### 2.8 Statistical Analysis

All experimental evaluations were repeated three times to ensure the accuracy and uniformity of the results. The resulting data were reported as mean with standard deviation to determine the degree of dispersion in the results. One-way analysis of variance (ANOVA) was used to assess statistical differences among groups. In this analysis, probability values (P-value) less than 0.05 were considered to indicate a statistically significant difference.

## 3 Results and Discussion

### 3.1 Characteristics of the Synthesized WL Nanoparticles

The XRD pattern of the synthesized WL nanoparticles is shown in Figure 2a. The position and intensity of the diffraction peaks are consistent with the standard pattern of this mineral phase, confirming that the WL particles were successfully synthesized with a well-defined crystal structure. The prominent peaks at angles of 25, 29, 32, 34, 40, and 49° indicate high crystallinity in the samples. The average crystallite size of WL was estimated to be approximately 17 nm using the Scherrer equation. The sharpness of these peaks also indicates a high degree of crystallinity. Furthermore, a close examination of the patterns revealed no signs of additional phases arising from changes in the concentrations of calcium or magnesium ions. This suggests that the synthesized particles were phase-pure.^34,36,43^ Therefore, XRD analysis indicates successful manufacturing of WL nanoparticles with a well-ordered structure and high purity.

**Figure 2:**
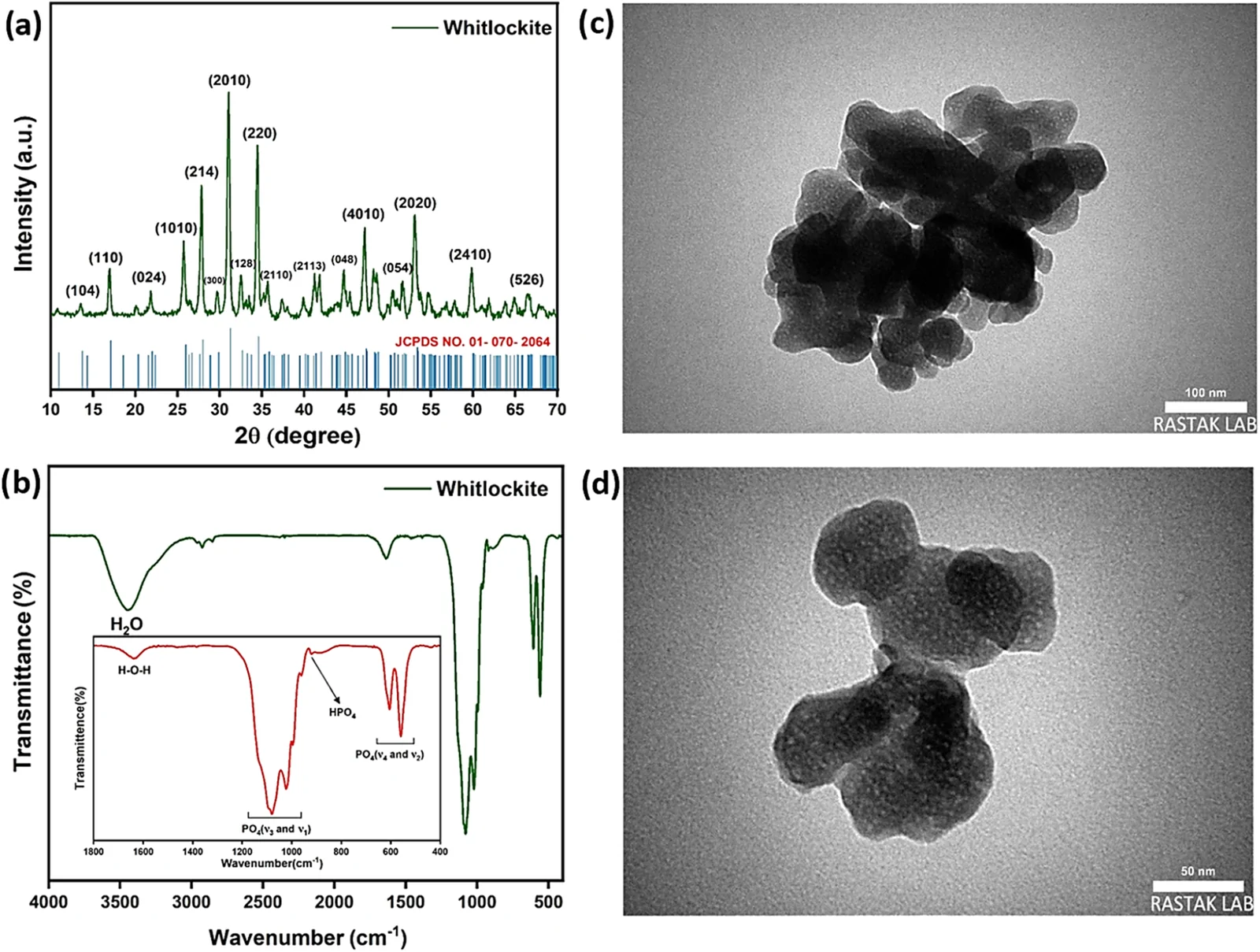
(a) XRD pattern, (b) FTIR analysis, (c, d) TEM image of synthesized WL nanoparticles powder.

Figure 2b presents the results of FTIR analysis to investigate the presence of active chemical groups in WL nanoparticles. The observation of an absorption peak at around 872 cm^–1^ is attributed to the presence of the HPO_4_^2–^ ion, a compound known as a specific structural feature of bone WL and distinguishing it from other calcium phosphate phases such as β-TCP or similar magnesium-containing CaP compounds. The absorption band observed at around 1029 cm^–1^ is also related to the asymmetric vibrations of the phosphate groups, while the peak in the 605 cm^–1^ region represents the bending vibrations related to the O–P–O bonds. The absence of bands characteristic of additional phases, including residual CaP in the 650 and 3570 cm^–1^ regions, is considered as an indication of the structural purity of WL.^32,40,43^ Also, the broad absorption band around 3500 cm^–1^ is likely attributed to hydroxyl groups adsorbed on the nanoparticle surface or to moisture trapped in the KBr used in sample preparation.

The TEM images of the WL nanoparticles are presented in Figure 2c,d. WL nanoparticles are observed with a clustered dispersion pattern and irregular appearance, so that they exhibit a structure similar to intertwined chains. Quantitative measurements from the TEM images yielded an average particle size of 55 ± 25 nm. The relatively large standard deviation indicates a broad particle-size distribution, while the clustered regions observed in the micrographs suggest that some nanoparticles formed partial agglomerates during synthesis and/or subsequent drying. This aggregation can reasonably be attributed to the tendency of nanoscale inorganic particles to form clusters during precipitation, drying, and subsequent processing.^43,44^ The morphological pattern is consistent with reports^26^ that implying the significant sensitivity of WL size and shape to Ca to Mg molar ratios. In particular, it was found that increasing the Mg content or reducing the relative Ca content can lead to a decrease in particle size and an increase in morphological uniformity.^26,32,36,43^ The small difference in the average particle size compared to previous studies^43,44^ is probably due to minor differences in the synthesis conditions or initial composition. The combination of XRD, FTIR, and TEM data provides clear evidence of the successful synthesis of WL nanoparticles.

Figure 3a presents the camera images of the 3D-printed scaffolds. The images of the scaffolds produced show regular, precise architectures, indicating good printing quality and the tunability of the PCL-based formulations. In the investigated compositions, the addition of Alg and WL nanoparticles did not have any obvious negative effect on the flow properties required for the layer deposition process.^15,22,45,46^ Also, no signs of significant distortion, warping, or geometric deviation were observed during printing, indicating good dimensional stability of the system during printing. This positive feature seems to be largely due to the gradual crystalline behavior and uniform thermal properties of PCL. Compared with polymers such as PLA or ABS, PCL prevents internal stresses and disruption of structural continuity because of its slower solidification rate and reduced tendency to shrink.^15,22,45,46^ Also, the slow crystallization of PCL in the interlayer region provides sufficient time for the polymer chains to rearrange before solidification, thereby increasing the adhesion strength of the layers. This phenomenon is attributed to the flexible nature of the molecular chains, a specific molecular weight, and a crystalline structure, which are well adapted to the printing conditions.^32,45,46^ Ultimately, the geometric stability of the structures after adding bioactive components such as WL and Alg confirms that printability and final scaffold quality are unaffected, and that the combined formulation still maintains high structural integrity.

**Figure 3:**
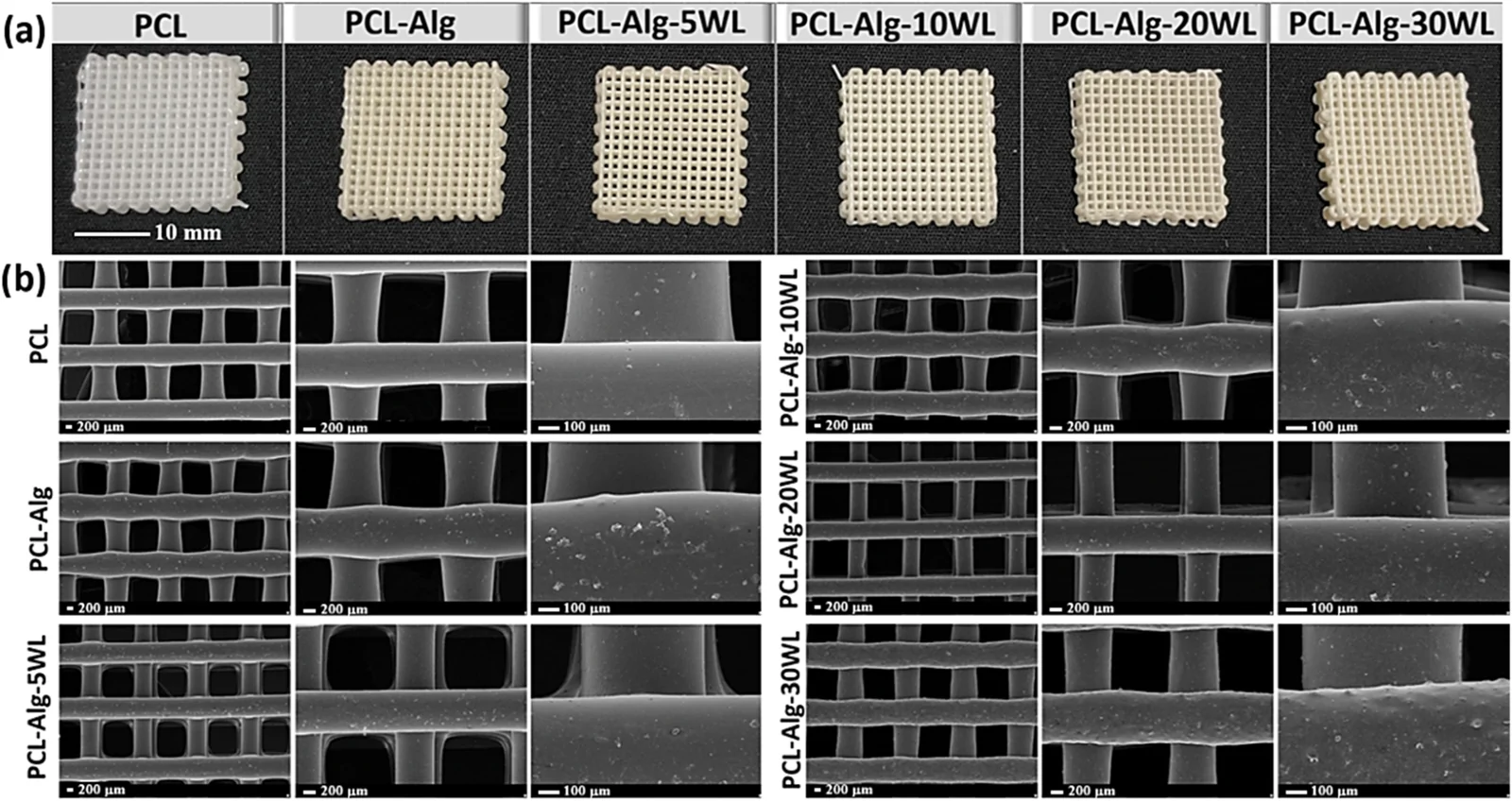
(a) optical images and (b) SEM images of 3D-printed PCL, PCL/Alg, and PCL/Alg-WL composite scaffolds containing 5, 10, 20, and 30 wt % WL.

SEM images of the 3D-printed PCL, PCL/Alg and PCL/Alg-WL scaffolds are shown in Figure 3b. The presence of a porous and interconnected network is clearly visible, a structure that is considered essential for effective nutrient transport, cellular penetration, waste removal, and new bone tissue formation. In the scaffold reinforced with WL nanoparticles, the pore size ranged between approximately 800 and 950 μm. Not only is this size range considered suitable for bone regeneration, but there was also no significant difference in pore diameter between the different groups. These findings indicate that the addition of WL did not disrupt the designed architecture of the scaffold, and the geometric integrity of the pores was maintained.^21,44^ Previous studies^1,2,47,48^ demonstrated that the addition of inorganic nanoparticles to biopolymers can alter their flow and extrusion properties; for example, some systems experience changes in filament diameter and thus pore size in response to nanoparticle loading. However, the current scaffolds demonstrated that the chosen combination of materials did not cause any undesirable changes during printing. A closer zoom reveals the structural details of the printed filaments. The filaments were smooth, uniform, and well-connected. Although some WL particles appeared on the surface, no evidence of severe clustering or architectural degradation was observed. The results confirm that this composite system not only exhibits good printability but also maintains its structural integrity and overall scaffold quality.

To further investigate the dispersion of WL nanoparticles in the composite matrix containing PCL and Alg, EDS elemental mapping analysis was used (Figure 4). In scaffolds fabricated from pure PCL and PCL/Alg, EDS images showed a uniform distribution of major elements such as carbon and oxygen on the surface of the fibers; the addition of Alg had no obvious effect on structural changes. The low sodium content in the PCL/Alg samples may be due to residual sodium alginate within the polymer network. In samples containing WL, EDS data clearly showed the key elements of this nanoparticle, including Ca, Mg, P, and O, throughout the composite structure. The uniform diffusion of signals of these elements along the scaffold fibers indicates a homogeneous distribution and a lack of local aggregation of nanoparticles. Slight differences in the intensities of some signals may reflect regions with varying concentrations of organic matter or carbon residues. The results indicate successful and uniform placement of WL in the composite polymer substrate, which can play an effective role in its biological performance.

**Figure 4:**
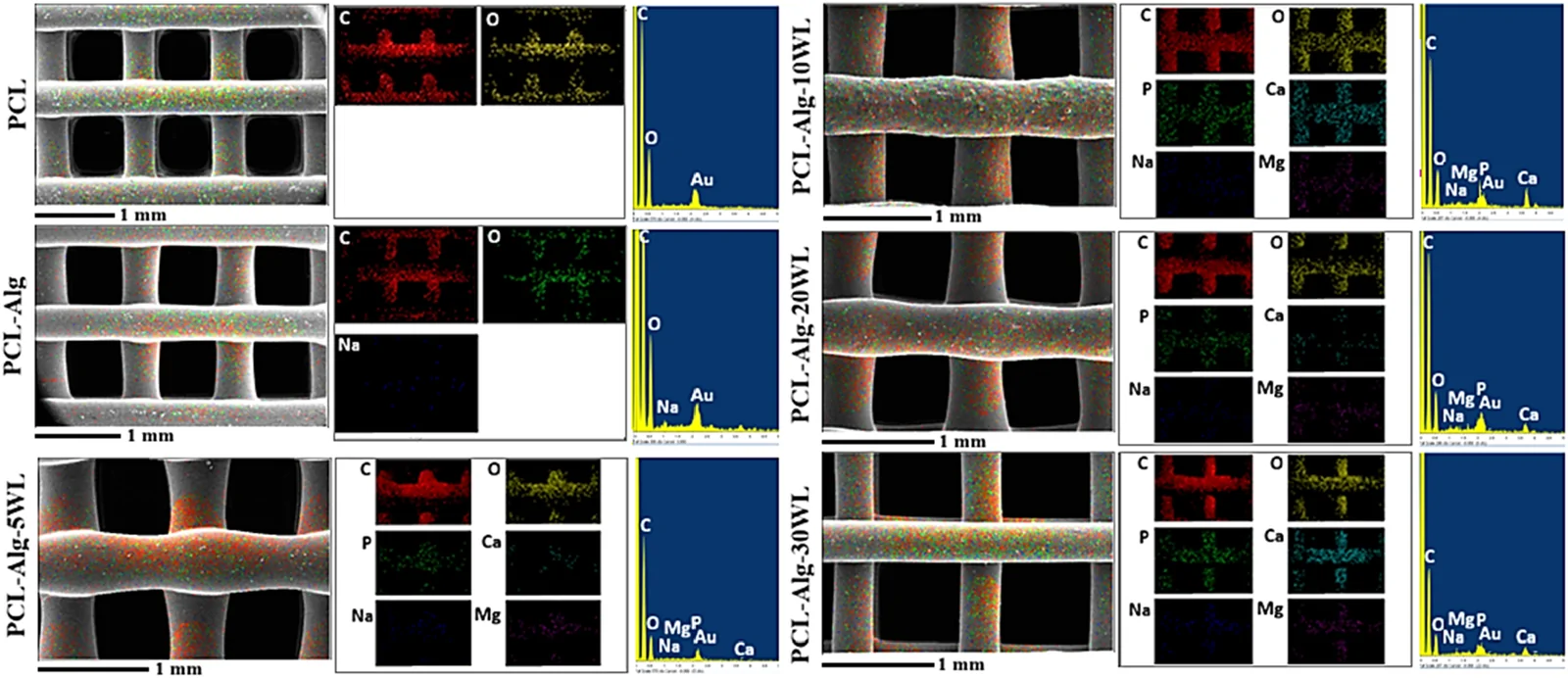
EDS elemental mapping of 3D-printed PCL, PCL/Alg, and PCL/Alg-WL composite scaffolds containing 5, 10, 20, and 30 wt % WL.

Figure 5a shows the XRD patterns of 3D-printed scaffolds prepared from PCL/Alg-WL scaffolds containing different WL concentrations. The pure PCL scaffold showed two distinct diffraction peaks at approximately 2θ = 21 and 23.7°, corresponding to the (110) and (200) crystal planes of PCL's orthorhombic structure, respectively. The incorporation of Alg into PCL did not significantly alter its crystallinity, as the characteristic PCL diffraction peaks remained essentially unchanged. The absence of distinct additional reflections attributable to Alg suggests that no separate crystalline Alg phase was resolved in the blend.^22,49^ The addition of WL nanoparticles to the polymer system resulted in additional diffraction peaks associated with the ceramic WL phase, confirming their successful incorporation into the PCL/Alg scaffold. The increasing intensity of these peaks with increasing WL concentration was clearly evident, indicating a higher content of crystalline components in the composite matrix.^26,36^ At the same time, a relative decrease in the intensity of the characteristic bands of PCL was observed after incorporation with WL, which could be attributed to the physical interaction between the polymer chains and the inorganic nanoparticles or to the minor effects of WL on the crystallization process of PCL.

**Figure 5:**
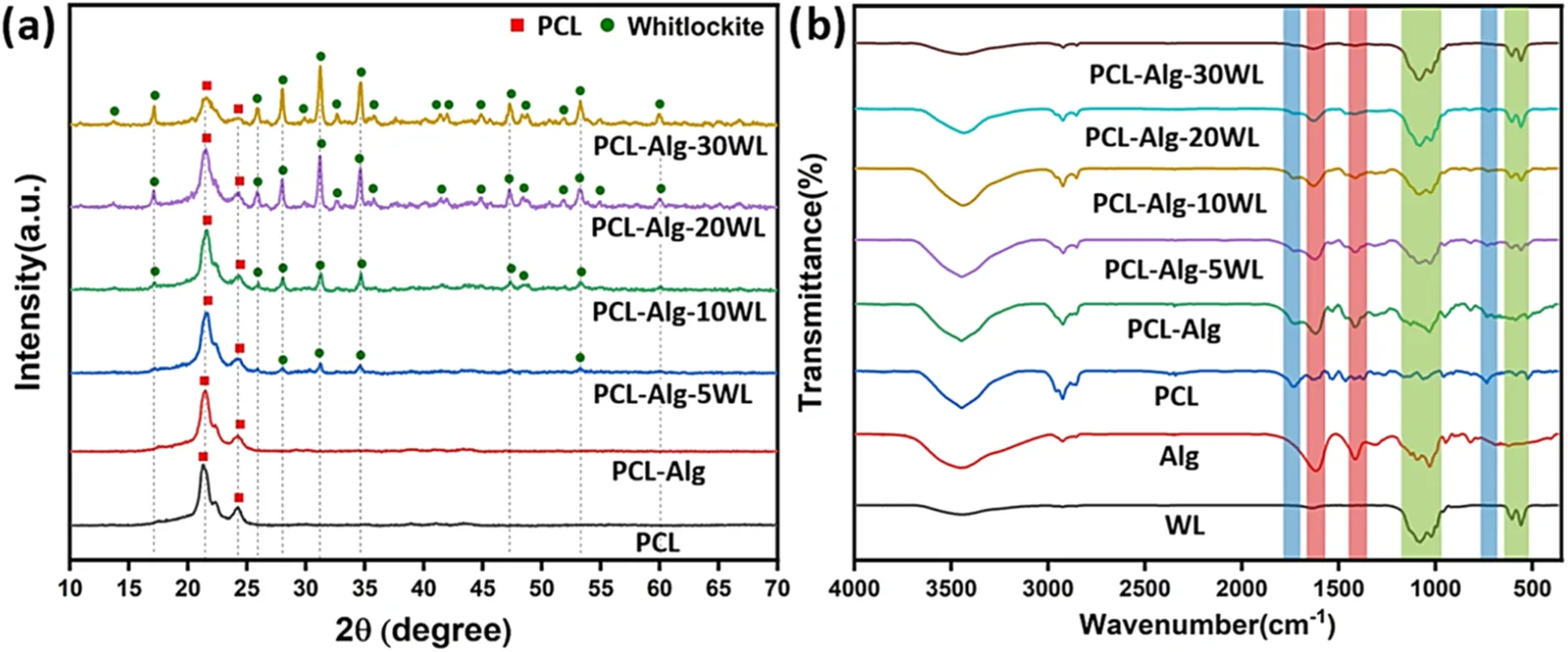
(a) XRD patterns, and (b) FTIR graphs of the WL, Alg and 3D-printed PCL, PCL/Alg, and PCL/Alg-WL composite scaffolds containing 5, 10, 20, and 30 wt % WL.

Figure 5b shows the FTIR spectra of the 3D-printed PCL/Alg-WL scaffolds with different WL contents. The characteristic band observed around 1722 cm^–1^ in the pure PCL sample is related to the stretching of the carbonyl bond in the ester groups. In contrast, the Alg spectrum shows peaks near 1602 and 1417 cm​^–1^, corresponding to the asymmetric and symmetric stretching vibrations of the carboxylate groups, respectively.^22,50,51^ In the PCL/Alg composite sample, a broad band appears in the range of 3200–3500 cm^–1^, which is related to the stretching of the hydroxyl groups; these groups play a key role in water adsorption and surface biointeractions. Also, bands are observed in the regions of 2940 to 2850 cm^–1^, which are aligned with the stretching vibrations of the C–H bonds of the hydrocarbon chains. The prominent peak in the region of 1735 cm^–1^ also refers to the ester bonds in the PCL structure.^22,50,51^ In addition, the absorption in the range of 1200 to 1100 cm^–1^ is related to the vibrations of the C–O bonds in the ester and ether structures found in both polymers.

The addition of WL nanoparticles to the composite scaffold structure led to the appearance of new absorption bands in the FTIR spectrum, which became more intense with increasing WL concentration in the scaffolds. These bands mainly indicate the presence of phosphate functional groups in the WL structure. In the region of 930 to about 1200 cm^–1^, signals were observed that were related to the asymmetric and symmetric stretching vibrations of the phosphate groups, indicating the successful integration of these groups into the composite matrix.^31,35,41,52^ Also, the presence of distinct bands in the range of 500 to 640 cm^–1^, in addition to the prominent peak at about 436 cm^–1^, is attributed to the bending vibrations of the phosphate groups, which further confirms the existence of the mineral phase of WL. In particular, the bands at 918 and 865 cm^–1^ indicate the presence of hydrogen phosphate groups (HPO_4_^2–^); this feature is considered as a specific marker and distinguishes the WL phase compared to other forms of calcium phosphate. Although the absorption at 865 cm^–1^ was relatively weak, the stronger band at 918 cm^–1^ clearly indicates the success of encapsulation of WL with the PCL/Alg scaffold.

Surface hydrophilicity is a key component in the design of bioscaffolds, as it directly contributes to basic biological processes such as cell adhesion, protein interactions, and initial cell-surface interactions.^23,53^ To analyze this property, the water absorption behavior and surface contact angle of the prepared scaffolds were measured, and the data are presented in Figure 6a,b. In pure PCL, the contact angle was measured at about 98°; this value indicates the hydrophobic nature of this polymer. This behavior is quite predictable considering the chemical structure of PCL and the lack of active polar groups on its surface, and is also in line with previous studies.^1,15^ Such surfaces typically exhibit limited interaction with the aqueous environment and show low initial adsorption capacity for biological fluids. In contrast, adding Alg to the composition clearly improved wettability, decreasing the contact angle to about 86°. This dramatic change is due to the presence of hydrophilic structures of Alg, especially functional groups such as hydroxyl and carboxylate, which can readily form hydrogen bonds with water molecules.^15,53,54^

**Figure 6:**
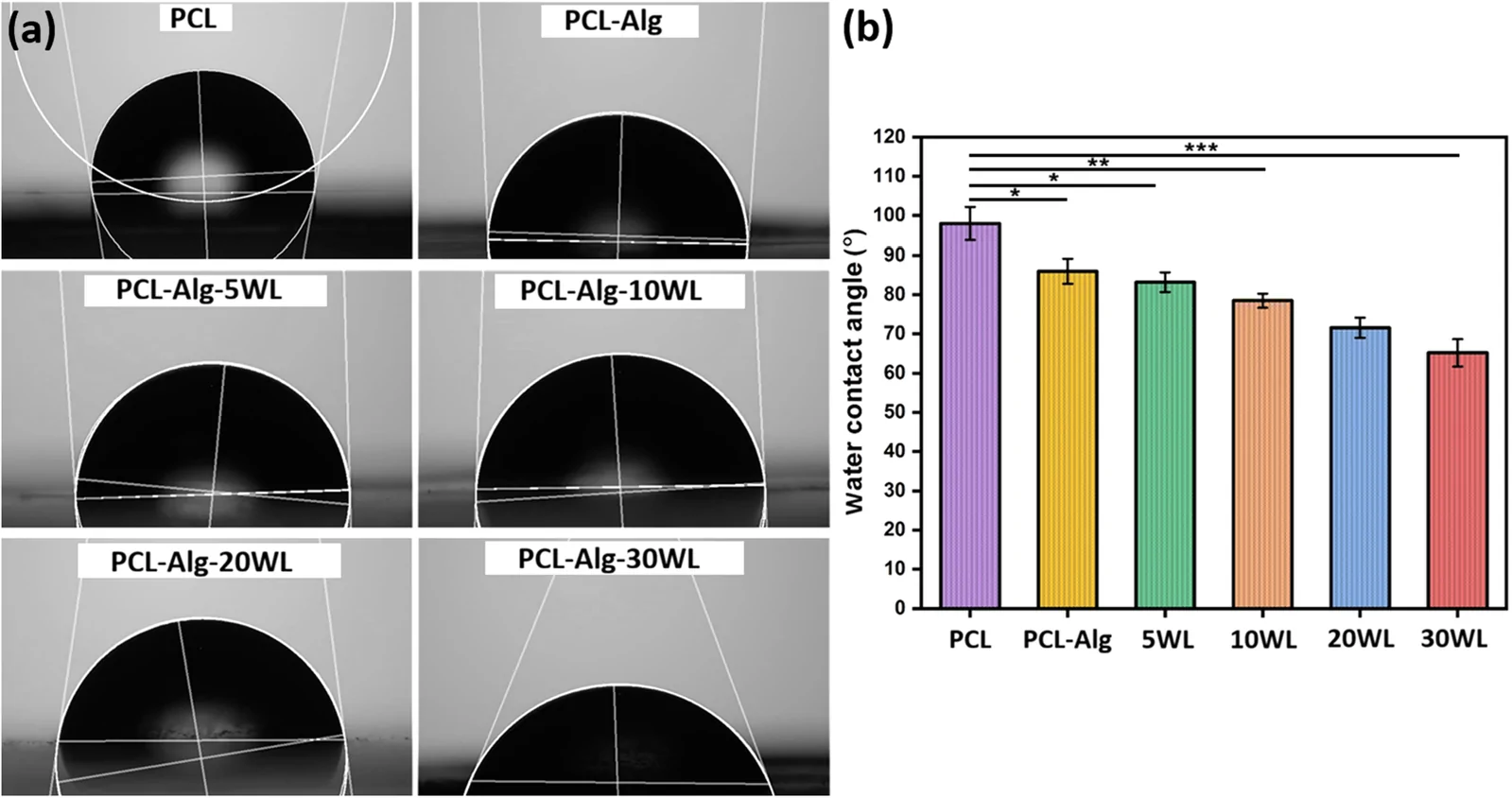
(a) Surface contact angle image and (b) contact angle value of the 3D-printed PCL, PCL/Alg, and PCL/Alg-WL composite scaffolds containing 5, 10, 20, and 30 wt % WL. Data are presented as mean ± SD (*n* = 3). Note: *p < 0.05, **p < 0.01, and ***p < 0.001.

Incorporating Alg into PCL scaffolds increased the responsiveness of these systems to biological aqueous environments, which could play a key role in improving liquid absorption, surface wetting, and early cell-material interactions. Importantly, the contact-angle results demonstrate that the progressive enhancement in wettability was predominantly associated with increasing WL content rather than with Alg alone. Following WL incorporation, the water contact angle decreased progressively from approximately 83° for 5WL to 78, 71, and 65° for 10WL, 20WL, and 30WL, respectively. Alg and WL incorporation significantly reduced the water contact angle compared with pure PCL (*p* < 0.05–0.001). This phenomenon was mainly due to the inherent hydrophilicity of ceramic materials and the surface chemistry of WL. In fact, increasing the WL content from 5 to 30 wt % increased the presence of active mineral phases on the scaffold surface, thereby expanding the contact area between the solid and liquid phases and decreasing the water contact angle.^1,34^ Also, the introduction of WL into the structure can create active surfaces including phosphate, calcium, and magnesium ions which exhibit high affinity for interacting with water, thereby enhancing surface hydrophilicity.^1^ The continuous decrease in contact angle after introducing Alg and then WL indicates a gradual improvement in the wettability of PCL-based scaffolds. These surface modifications not only facilitate biomolecule binding and adsorption but also provide a basis for more favorable cell colonization and enhanced scaffold-host tissue interaction, a key factor in effective bone regeneration.

### 3.2 Mechanical Properties

To support tissue regeneration, a bioengineered scaffold must exhibit mechanical and elastic properties that closely match those of the target tissue. This adaptation ensures the scaffold maintains its geometric shape while providing the mechanical stability needed to withstand physiological forces during tissue regeneration.^19,22^ The stress–strain curves are presented in Figure 7a, while the quantitative compressive properties are summarized in Figure 7b–d. The pure PCL scaffold showed a compressive strength of about 7 MPa. Introducing Alg into the scaffold increased the compressive strength to about 14 MPa, likely due to increased network cohesion and improved stress tolerance provided by this biopolymer.^15,19,22^ Interestingly, adding 5 wt % WL nanoparticles to the PCL/Alg composite further enhanced the mechanical properties, with the strength reaching approximately 22 MPa. This observation suggests that WL at this ratio can serve as an effective reinforcing role and enhance the compressive stability of the assembly by improving the force transfer at the interfaces between the organic and inorganic phases. However, further increases in WL content reduced the compressive strength to approximately 14.5, 14.3, and 12 MPa for 10WL, 20WL, and 30WL, respectively. This performance loss may be due to nanoparticle aggregation and the formation of stress concentration zones, which hindered uniform load transfer and disrupted interphase bonding. Excessive particle loading may also reduce the thickness of the printed filaments and weaken the scaffold’s overall structure.^32,34,37,39^ These results indicate that the mechanical response of PCL/Alg-WL scaffolds is composition-dependent. The presence of Alg alone improves mechanical stability, but the addition of an optimal amount of WL further enhances scaffold strength. Among the scaffolds examined, the PCL/Alg-5WL composite showed the best balance between the properties of flexibility, and structural integrity, supporting its potential for bone tissue engineering.

**Figure 7:**
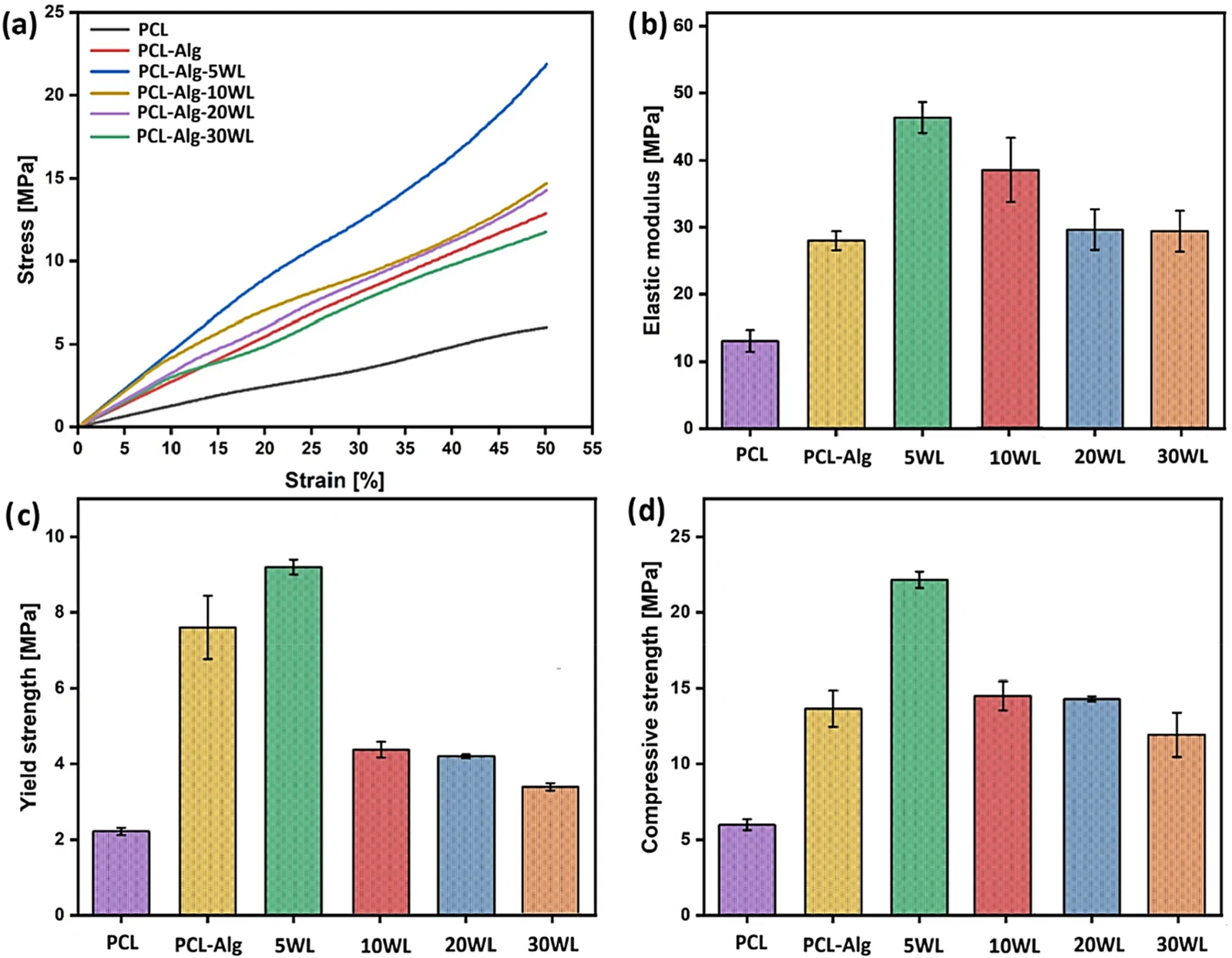
(a) Strain–stress compressive curve, (b) elastic modulus value, (c) yield strength value, and (d) compressive strength value of 3D-printed PCL, PCL/Alg, and PCL/Alg-WL composite scaffolds containing 5, 10, 20, and 30 wt % WL.

### 3.3 Apatite Formation

The formation of apatite structures on the surfaces of the scaffolds was evaluated using scanning electron microscopy after a 28-day immersion period in a pseudophysiological solution. As shown in Figure 8a, increasing WL content promoted mineralization. While pure PCL scaffolds showed no obvious precipitation, the WL-containing composite scaffolds exhibited progressively more pronounced apatite-like deposition. A significant similarity was observed between the groups with 5 and 10 wt % WL in terms of precipitation intensity, and this trend was also repeated in formulations with higher amounts, i.e., 20 and 30 wt % WL. This pattern indicates that WL significantly enhances the bioactive behavior of polymer-ceramic systems. The increased hydroxyapatite crystallinity can be attributed to surface activation and the formation of nucleation sites through ionic interactions. Upon contact with the simulated environment, polymer surface interactions exposed polar groups such as hydroxyl and carboxylate.^1,48^ These chemical groups, by absorbing Ca^2+^ ions, created favorable conditions for the precipitation of phosphate ions, and apatite nuclei eventually formed on the surface. Continuing this process with longer immersion times led to more active sites and, consequently, increased apatite growth.^1,48^

**Figure 8:**
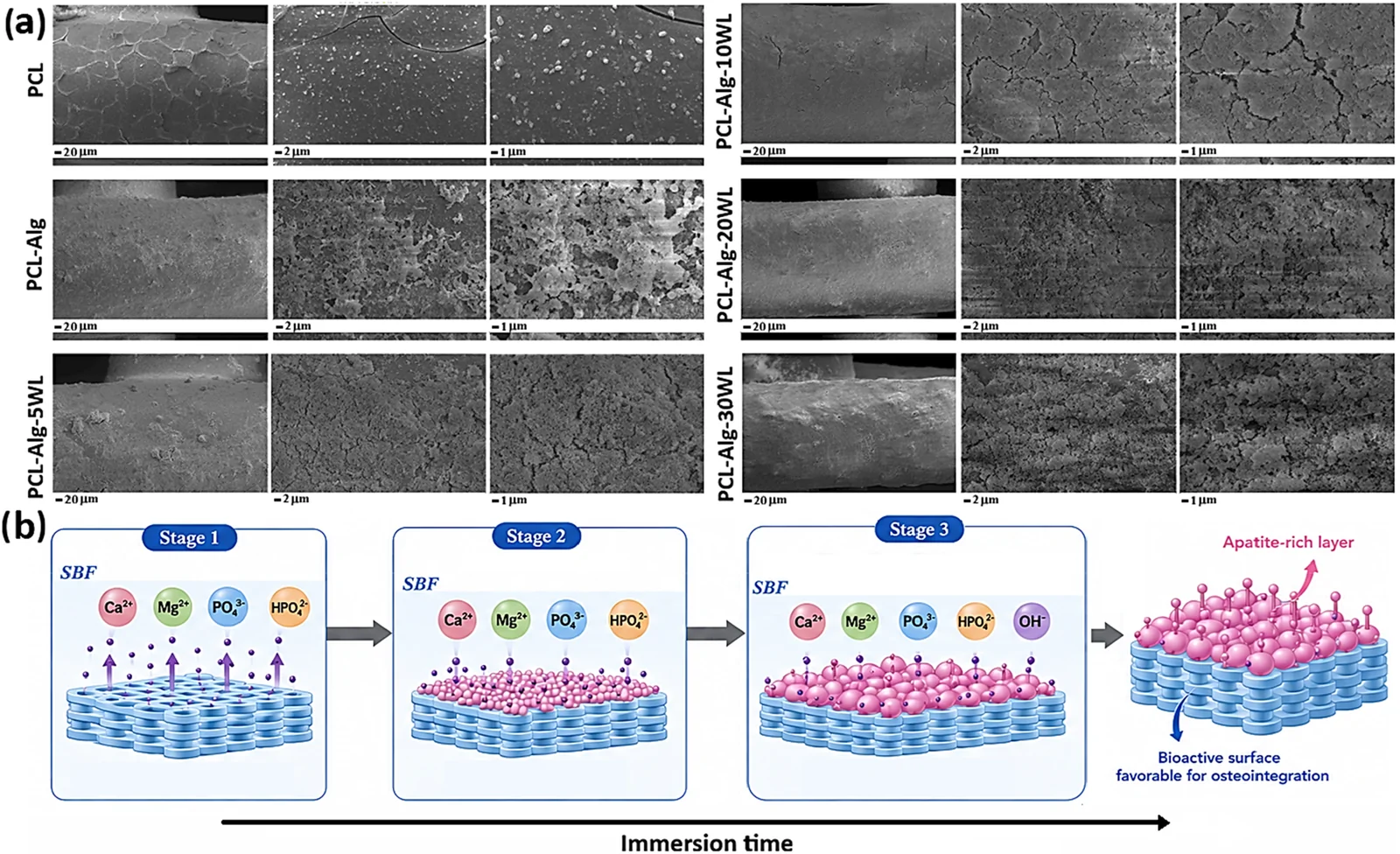
(a) SEM images of PCL, PCL/Alg, and PCL/Alg-WL composite scaffolds containing 5, 10, 20, and 30 wt % WL after immersion in SBF at various magnifications, and (b) Apatite formation mechanism on the surface of PCL/Alg scaffolds containing WL after soaked in SBF for 28 days.

By adding WL nanoparticles to the scaffold system, calcium and magnesium phosphate-rich structures are introduced into the matrix, thereby enhancing the bioactive properties of the scaffold surface. The ions released from WL, especially Ca^2+^, Mg^2+^ and phosphate species, provide local saturation conditions around the scaffold and create a suitable substrate for hydroxyapatite nucleation processes. The presence of Mg^2+^ can also be effective in regulating the mineralization balance and help stabilize the apatite structures on the surface.^1,52^ Therefore, the more extensive apatite deposition observed in the WL-containing scaffold is likely the result of a combination of several factors: chemical activation of the scaffold surface, increased release of key ions, and the inherent biocompatible nature of these nanoparticles.^41,43^ Such ion-mediated mineralization may provide a surface environment favorable for subsequent osteogenic cellular responses, as reported for whitlockite-containing biomaterials. Previous studies^34,37,38^ demonstrated the presence of WL in scaffolds, with both in vitro and in vivo evaluations. WL may promote more effective bone formation and provide a more favorable balance between structural stability and absorption rate than other ceramic fillers such as HAp. This result indicates the key role of WL in enhancing the bioactivity of PCL-based scaffolds and introduces it as an effective mineral phase in the osteogenesis process.^27,28,34^ Based on the present mineralization results, the 5WL formulation already provides a marked enhancement in apatite-forming ability compared with the WL-free scaffold, while further increases in WL content produce comparatively smaller incremental changes.

The mechanism of apatite formation on the surface of 3D printed PCL/Alg-WL scaffolds after soaking in SBF is exhibited in Figure 8b. Upon immersion in SBF, the scaffolds undergo a sequential mineralization process, indicating their bioactive properties. In the first step, the WL nanoparticles embedded in the scaffold slowly release Ca^2+^, Mg^2+^, PO_4_^3–^, and HPO_4_^2–^ ions into the surrounding environment, thus creating a localized region with a high concentration of these ions around the scaffold surface. This supersaturation condition provides the environment for the initiation of nucleation of HAp crystals in the second step, leading to the formation of an initial layer of mineral deposits on the scaffold surface. Subsequently, the role of Mg^2+^ ions becomes more prominent; the sustained presence of these ions stabilizes the CaP mineral phases and, by delaying premature crystallization, allows for a more uniform and extensive deposition (stage 3). In subsequent stages, this initial layer gradually grows and becomes a continuous and poorly crystalline layer of apatite, which structurally closely resembles the natural mineral phase of bone. Such a surface not only provides a suitable substrate for the attachment of osteogenic cells, but also can facilitate osteointegration processes in terms of biological function. In the meantime, WL does not only play a role as a mechanical reinforcement, but also acts simultaneously as an active ionic source and regulates and enhances the initiation and spread of surface mineralization by continuously releasing bioactive ions. This ionic dynamics plays a significant role in enhancing the overall bioactivity of the scaffold and its potential in bone tissue regeneration.

To more accurately assess the biological behavior of the scaffolds, apatite deposition on the surface of the scaffolds was examined after immersion in an SBF solution. In pure PCL scaffolds, there was hardly any sign of mineral coating. Only limited spots of mineral accumulation were observed, which EDS analysis confirmed to contain calcium and phosphorus, indicating the initial formation of a calcium phosphate phase (Figure 9). However, the amount of these deposits remained insignificant; a situation that is fully consistent with the hydrophobic nature and lack of surface-active interaction of PCL with the biological environment. The introduction of Alg into the PCL scaffold structure led to a significant increase in surface deposits. This phenomenon can be attributed to improved surface wettability, as previously confirmed by a decrease in the surface contact angle. Due to the hydrophilic nature of Alg, the interaction between the scaffold surface and the liquid medium was likely increased, facilitating ion exchange and, consequently, the initial mineralization process. The functional groups present in Alg can make the surface more suitable for cation adsorption and apatite deposition.^22,48^ The incorporation of WL nanoparticles significantly increased the amount and density of mineral deposits. The formation of a dense layer of apatite-like crystals on the surface of these scaffolds is evidence of high bioactivity and creating a suitable mineral substrate. It seems that WL accelerated the nucleation and growth of apatite by providing active and biocompatible sites.^41,43^

**Figure 9:**
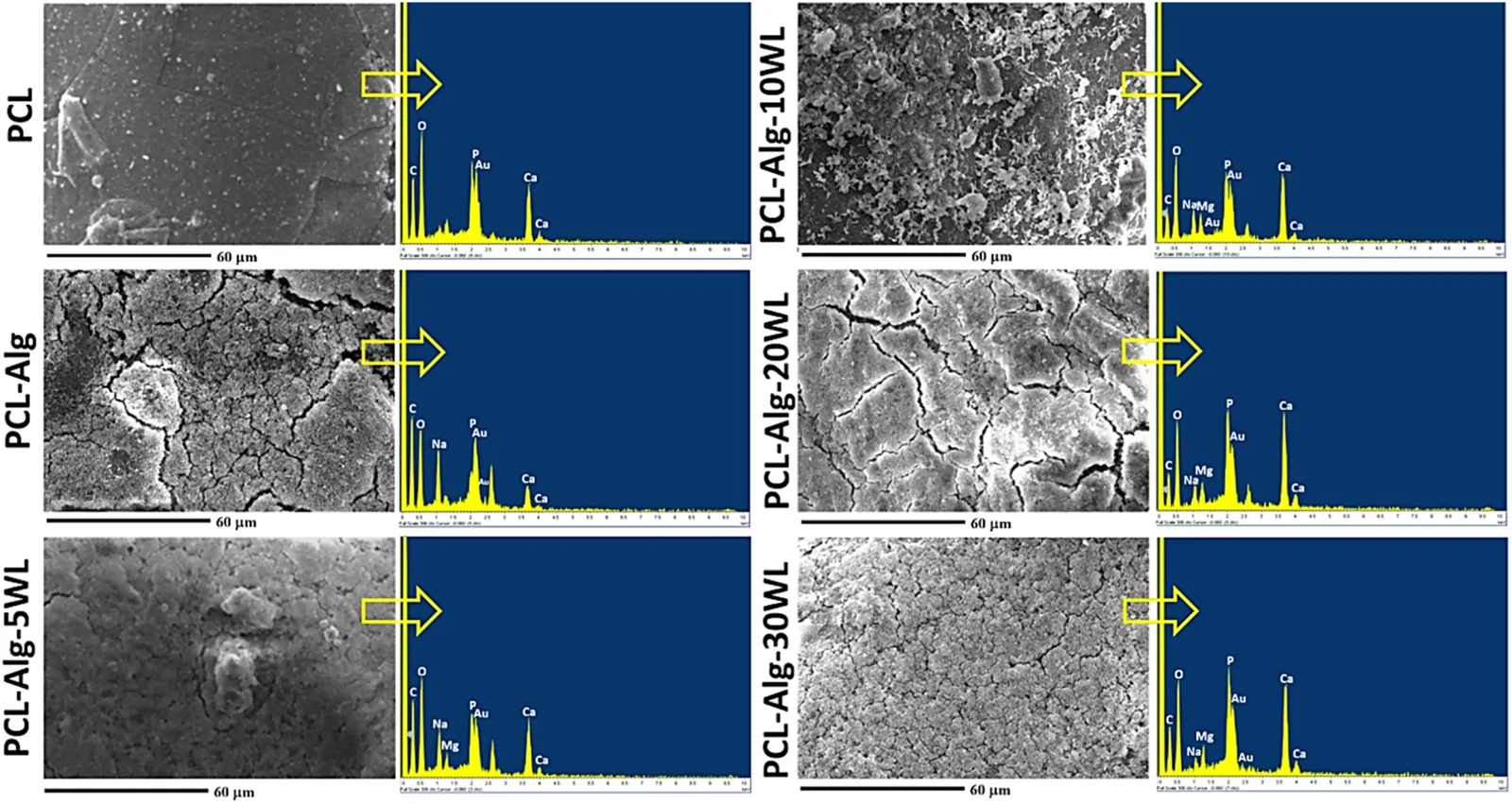
SEM images and corresponding EDS analysis of PCL, PCL/Alg, and PCL/Alg-WL composite scaffolds containing 5, 10, 20, and 30 wt % WL after 28 days of immersion in SBF.

The EDS results indicated that calcium and phosphorus are present in the surface deposits of the scaffolds, which is considered a reason for the formation of an apatite-like mineral layer on their surfaces. The increased bioactivity of scaffolds reinforced with WL is likely due to the direct role of this ceramic phase in intensifying surface mineralization. The chemical composition of WL, which contains bioactive elements such as Ca, Mg, and P, provides a high potential for creating desirable biological responses. During the placement of scaffolds in SBF, ions released from WL nanoparticles in the peripheral area of the surface cause a local increase in the concentration of ionic species such as Ca^2+^, Mg^2+^, and phosphate. This local supersaturation provides a suitable environment for the initiation of apatite crystal nucleation. The signals recorded by EDS, including calcium, phosphorus and oxygen, indicate this process. In addition, the presence of divalent ions such as Ca^2+^ and Mg^2+^ can accelerate the stable growth of the mineral layer and prevent its dissolution by affecting the solution equilibrium conditions.^34,43,48^ Therefore, WL can be considered not only a structural reinforcement but also an active phase in the release of bioactive ions, which simultaneously facilitates deposition and mineralization and ultimately enhances scaffold viability.

### 3.4 Biodegradation of 3D-Printed Scaffolds

For successful guided tissue regeneration, it is necessary that the scaffold used degrades at an appropriate rate, so that it maintains its mechanical support role until the new tissue is fully formed and then gradually gives way to the newly formed tissue.^8,34^ In this regard, the degradability of the PCL/Alg-WL scaffolds was evaluated over 56 days of immersion in PBS solution (Figure 10a). The results showed that, although all scaffolds experienced weight loss, the extent of this loss varied with their composition. The pure PCL scaffold showed the least weight change, losing only 2.78% of its initial mass after 8 weeks. This slow degradation behavior is attributed to the hydrophobic nature of PCL, as limited water penetration into the polymer matrix greatly reduces hydrolysis. In contrast, the PCL/Alg composite scaffold experienced a greater weight loss of 8.58%, indicating the effective contribution of Alg in water absorption and accelerated scaffold degradation.^34,48^ Adding WL nanoparticles to the composition significantly increased the weight loss of the scaffolds, which was directly proportional to the WL loading. The sample containing 30 wt % WL exhibited the highest weight loss, 12.24%. This phenomenon seems to result from two synergistic mechanisms: first, WL, by increasing hydrophilicity, facilitates liquid penetration into the scaffold and accelerates degradation reactions; second, as the ceramic particles gradually leave the scaffold during immersion, microporous structures are formed on the surface and inside the scaffold, which increase the surface contact with the aqueous environment and intensify the degradation process.^41,42^

**Figure 10:**
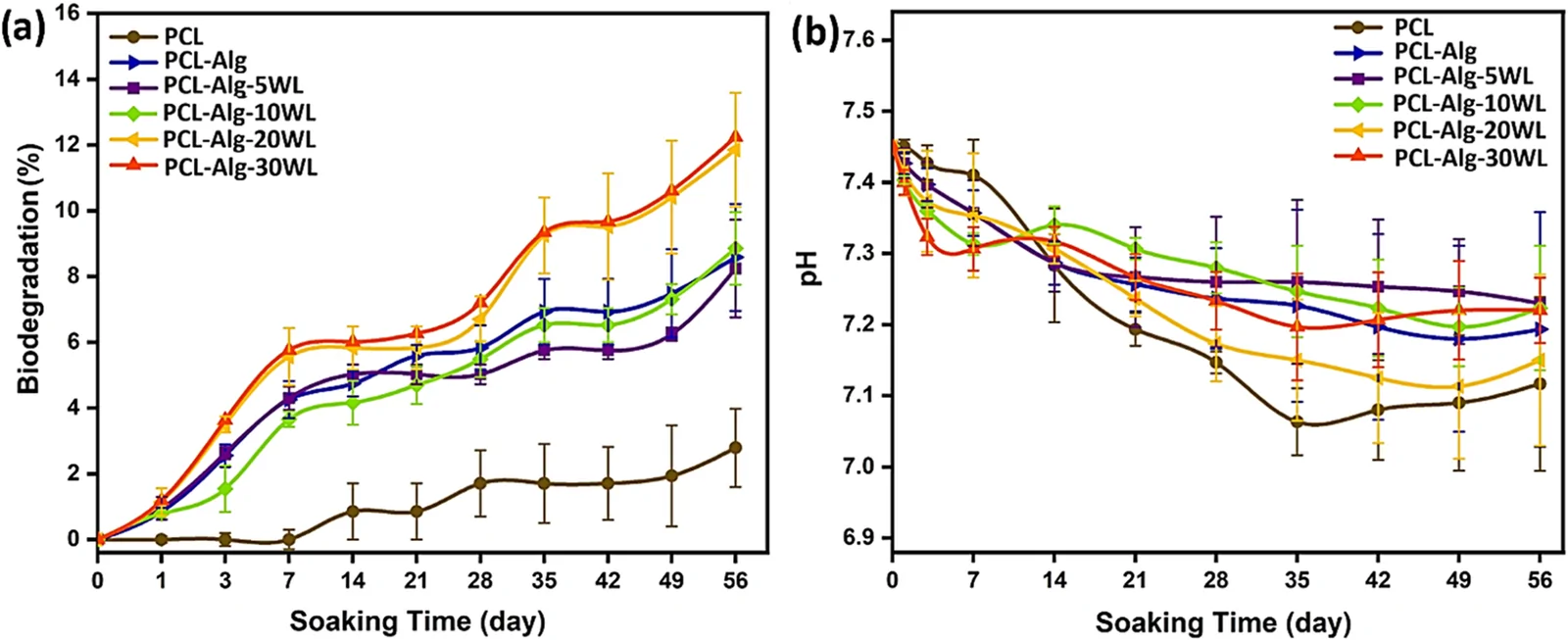
(a) degradation rate and (b) pH changes of 3D-printed PCL, PCL/Alg, and PCL/Alg-WL composite scaffolds containing 5, 10, 20, and 30 wt % WL after immersion in PBS for 56 days.

The increased degradation of WL-containing scaffolds can be considered a direct result of the interaction of the ceramic phase with the polymer substrate. Unlike aliphatic polymers such as PCL, which are inherently hydrophobic, ceramic particles exhibit a high tendency to absorb water due to the presence of polar groups on their surface. This surface feature leads to increased wettability, reduced contact angle, and ultimately accelerated moisture absorption and hydrolytic degradation processes. However, scaffold degradation depends not only on the types of constituent materials. Factors such as the surrounding environment, the molecular weight of the polymer, the degree of crystallinity, the degree of porosity, and the distribution of inorganic particles within the matrix all play a role in the degradation rate.^45,46^ Figure 10b shows the pH changes of the PBS solution after PCL/Alg-WL scaffold immersion. An insignificant decrease in the pH value was observed at the end of the 56-day immersion period. This decrease is attributed to the gradual release of acidic degradation products resulting from hydrolysis of the PCL ester bonds. In particular, carboxylic acid species, including hydroxycaproic acid, can gradually accumulate in the surrounding medium and contribute to a localized reduction in pH.^45^ The role of WL in this process is also significant; by facilitating water penetration and accelerating matrix degradation, not only is the decomposition process intensified, but also the rate of release of these acidic products is increased. As a result, WL particles, in addition to functioning as a bioactive reinforcing phase, can also help lower the pH of the surrounding environment by intensifying degradation.

### 3.5 Cell Responses

Biocompatibility is considered one of the key requirements in the design of tissue engineering scaffolds, as their potential toxicity needs to be carefully assessed before any in vivo use or preclinical testing.^34^ In this regard, the MTT assay was used to investigate the survival of MG-63 cells on different scaffolds after 1, 3, and 7 days of cell culture (Figure 11a). Based on the data, except for the pure PCL scaffold, all other scaffolds showed cell survival rates higher than 80%, indicating their favorable biocompatibility. In contrast, the PCL-only sample had the lowest viability rate among the groups, which is related to its hydrophobic and low surface area with the environment. The addition of Alg into the PCL scaffold improved the cellular response. This improvement can be attributed to the more hydrophilic nature, which increases surface hydration and wettability and provides a more suitable substrate for cell adhesion and growth. Furthermore, the swelling of Alg during attachment can increase its surface area, thereby enhancing cell adhesion and promoting cell viability.^15,16,22^

**Figure 11:**
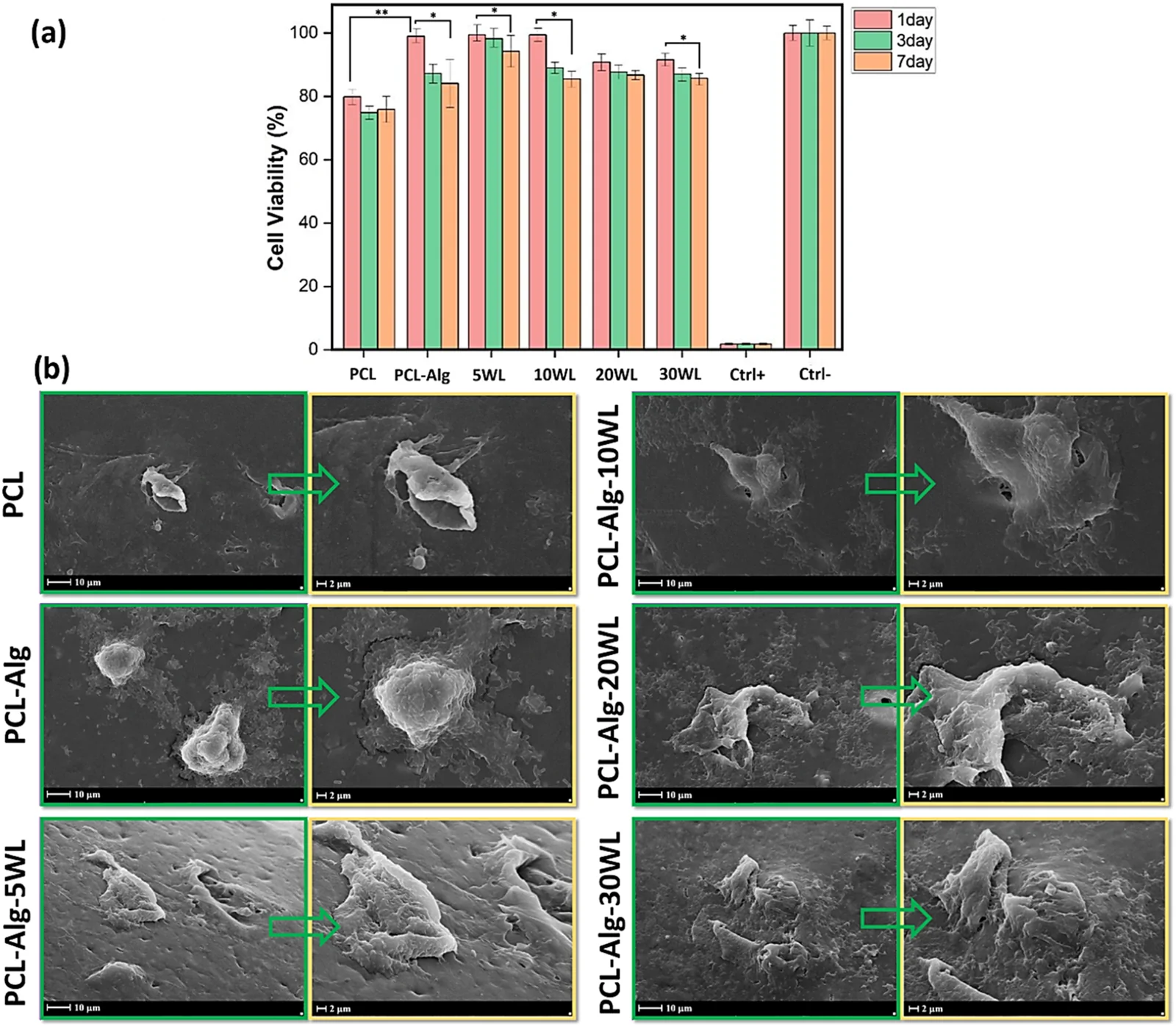
(a) cell viability results obtained from MTT assay, and (b) SEM micrographs of MG 63 cells cultured on the 3D-printed PCL, PCL/Alg, and PCL/Alg-WL composite scaffolds containing 5, 10, 20, and 30 wt % WL. Statistical analysis was performed by one-way ANOVA (*n* = 3, *P* < 0.05 (*), and *P* < 0.001 (**)).

By adding WL into PCL/Alg scaffolds, an escalation in the viability of MG-63 cells was observed. This phenomenon can be attributed to the release of ions such as Ca^2+^ and Mg^2+^, which effectively stimulate cell growth, adhesion, and metabolic activity. In addition, the calcium–magnesium phosphate-rich chemical structure of WL and the possibility of cross-linking interactions in the scaffold structure can provide favorable conditions for cell–substrate interactions.^28,41,42^ Notably, the scaffold containing 5 wt % WL showed the highest viability rate on day 7. This finding suggests that such a composite was able to establish a good balance between biological properties, surface characteristics, and cellular response. Also, negatively charged surface of WL facilitates protein adsorption, which in turn promotes better cell adhesion and proliferation in the early stages.^35,42^ Notably, the 5WL scaffold exhibited the highest cell viability on day 7, indicating that an intermediate WL content provided the most favorable overall cellular response among the investigated formulations. This result suggests that cellular performance depends on a balance among wettability, surface chemistry, mineral content, and cell–material interactions rather than surface hydrophilicity alone.

In the field of tissue engineering, the initial attachment of cells to the scaffold is of great importance, as this attachment drives subsequent processes, including proliferation, differentiation, and tissue regeneration. From this perspective, the scaffold functions similarly to the natural extracellular matrix and should provide the necessary environmental conditions for cell attachment and activity.^18,22^ SEM images of MG-63 cell attachment on the pure PCL scaffold surface revealed a limited level of cell adhesion (Figure 11b). Few cells were attached to the surface of this scaffold, and a stable and strong bond between the cell and the substrate was not formed. This can be attributed to the surface properties of PCL, which is hydrophobic and lacks active functional groups, and has little tendency to interact with biomolecules.^22,55^ In contrast, the Alg-modified scaffold showed significantly better cell attachment. MG-63 cells on this substrate formed lamellipodia- and filopodia-like structures, establishing more extensive interactions with the scaffold surface, indicating better biocompatibility and acceptance of this compound.

The improvement in cell adhesion can be largely attributed to the presence of Alg; this biomaterial, by increasing its hydrophilicity and introducing negatively charged groups, provides a suitable substrate for the initial adsorption of proteins. Such conditions make the scaffold surface more suitable for initial cell attachment, proliferation, and activity.^50,55^ Furthermore, the combination of WL with the PCL/Alg system significantly increased cell adhesion and spread. Cells in contact with PCL/Alg-WL scaffolds not only are attached more easily, but also showed a greater surface spread.^56–59^ This behavior is probably due to the presence of calcium–magnesium phosphate compounds present in WL; compounds that are chemically similar to the mineral structure of bone and therefore create a more bioactive environment for the adhesion of osteoblast-like cells.^41,42,52^ Another potential consequence of adding WL nanoparticles is increased surface roughness, which can provide more contact areas for cells and improve their mechanical adhesion to the scaffold. Also, the decrease in the contact angle of the water droplet on the surface indicates an improvement in wettability, which provides the necessary conditions for better cell attachment and spreading. The WL containing surface processes play a key role in early biological events after implantation, including cell-scaffold interactions, proliferation, and organization of newly formed tissue.^27,56,57^ Previous studies^28,34^ reported that WL, compared with HAp, can promote more favorable cellular responses while exhibiting no significant cytotoxicity. The presence of magnesium in the WL structure may be an important factor in enhancing the potential for osteogenic initiation.

## 4 Conclusion

In this study, a novel bioactive scaffold was designed and fabricated by incorporating WL nanoparticles at different percentages into a 3D-printed PCL/Alg scaffold. Results showed that the inclusion of WL in the PCL/Alg scaffold had no significant effect on the pore size and morphology. Among the studied formulations, 5 wt % WL provided the greatest mechanical reinforcement, resulting in the highest compressive strength. However, increasing WL beyond this level reduced the scaffold’s mechanical performance. In terms of biological functionality, WL led to a significant improvement in the scaffold’s performance. The scaffolds incorporating WL showed a significant enhancement in surface wettability and degradability, which can be attributed to the combination of Alg’s hydrophilic properties and WL’s ion-mediated dissolution behavior. From a bioactive perspective, the scaffolds incorporating WL exhibited significant apatite formation, confirming that bioactive ions released from WL effectively increased the surface tendency to mineralize. Based on cell culture results, the PCL/Alg scaffold containing 5% WL exhibited the highest levels of cell viability and adhesion. The improvement in cellular function is likely due to the combined effects of improved wettability, the release of active mineral ions, and surface chemistry that more closely resembles that of the inorganic phase of native bone. Taken together, the evidence suggests that encapsulating 5 wt % WL within the PCL/Alg scaffold can achieve a favorable balance between structural integrity and biological activity. This optimized formulation therefore represents a promising candidate for bone tissue engineering applications.

## Supplementary Material



## Data Availability

Data sets supporting the findings of this study are available from the corresponding author upon reasonable request.

## Notes

Compositions of the PCL/Alg-WL scaffolds and the corresponding WL contents (Table S1) and chemical composition of the SBF used for the mineralization study (Table S2) (PDF)
