# Supplementary material for: 3D-Printed Polycaprolactone/Alginate-Whitlockite Composite Scaffolds for Bioactive Bone Tissue Engineering
Source: ACS Mater Au. 2026 Oct 8;6(6):1540–55. doi: 10.1021/acsmaterialsau.6c00160 (PMC13760627; doi:10.1021/acsmaterialsau.6c00160)
Supplement: Supplementary file 1 [file mg6c00160_si_001.pdf]

3D-Printed Polycaprolactone/Alginate-Whitlockite Composite Scaffolds for Bioactive Bone Tissue Engineering

Sura Ali Al-nidawi[1], Aliakbar Najafinezhad[1], Hossein Abbastabar Ahangar[2], Hamid Reza Bakhsheshi-Rad[1,3,4,*], Jaroslaw W. Drelich[3,*]

[1] Department of Materials Engineering, Na.C., Islamic Azad University, Najafabad, Iran
[2] Department of Chemistry, Na.C., Islamic Azad University, Najafabad, Iran
[3] Department of Materials Science and Engineering, Michigan Technological University, Houghton, MI 49931, USA
[4] Faculty of Mechanical Engineering, Universiti Teknologi Malaysia, 81310 UTM Johor Bahru, Johor, Malaysia

***Correspondence:** rezabakhsheshi@gmail.com (H.R.B.-R.); jwdrelic@mtu.edu (J.W. D.)

Supporting Information

1. Fabrication of 3D-printed PCL/Alg-WL scaffolds

In this study, multicomponent PCL/Alg-WL scaffolds were fabricated using a desktop 3D bioprinter (Chakad, Iran) through a melt-extrusion-based additive manufacturing approach, as illustrated in Fig. 1b. A computer-aided design/computer-aided manufacturing (CAD/CAM) strategy was employed to construct the three-dimensional scaffold architecture through controlled layer-by-layer deposition. The total mass of each formulation was maintained at 5.00 g, while the Alg content was fixed at 0.5 g, corresponding to 10 wt% of the total formulation. WL nanoparticles were incorporated at 0, 5, 10, 20, and 30 wt% relative to the total PCL/Alg/WL formulation, corresponding to WL masses of 0, 0.25, 0.5, 1, and 1.5 g, respectively. Accordingly, the PCL contents were adjusted to 4.5, 4.25, 4, 3.5, and 3 g, respectively, as summarized in Table S1.

Table S1. Composition of the PCL/Alg-WL scaffold formulations used for 3D printing.

Formulation	PCL (g)	Alg (g)	PCL + Alg (g)	WL (g)	WL wt.% of total
PCL/Alg	4.50	0.50	5.00	0.00	0
PCL/Alg-5WL	4.25	0.50	4.75	0.25	5
PCL/Alg-10WL	4.00	0.50	4.50	0.50	10
PCL/Alg-20WL	3.50	0.50	4.00	1.00	20

PCL/Alg-30WL	3.00	0.50	3.50	1.50	30

2. Mineralization examination

To investigate the biomineralization potential of the scaffolds, the samples were incubated in 10 mL of SBF for 28 days under controlled temperature conditions (37°C) with chemical composition as listed in Table S2. The simulated body fluid (SBF) was prepared according to the method described by Kokubo and Takadama [1], with an ionic composition comparable to that of human blood plasma. After this period, the scaffolds were removed from the reaction medium and rinsed with distilled water upon removal from the SBF solution, followed by drying in an oven at 40 °C for 24 h.

Table S2. Ionic composition of Kokubo simulated body fluid (SBF) in comparison with human blood plasma.

**Solution**	**Ion concentration (mmol/L)**							
	**Na^+**	**K^+**	**Ca^{2+}**	**Mg^{2+}**	**HCO_3^-**	**Cl^-**	**HPO_4^{2-}**	**SO_4^{2-}**
Plasma	142.0	5.0	2.5	1.5	27.0	103.0	1.0	0.5
Kokubo (c-SBF)	142.0	5.0	2.5	1.5	4.2	147.8	1.0	0.5

[1] Kokubo T, Takadama H. How useful is SBF in predicting in vivo bone bioactivity?. Biomaterials. 2006;27(15):2907-15.
